# Epigenetic Changes with Dietary Soy in Cynomolgus Monkeys

**DOI:** 10.1371/journal.pone.0026791

**Published:** 2011-10-25

**Authors:** Timothy D. Howard, Shuk-Mei Ho, Li Zhang, Jing Chen, Wei Cui, Rebecca Slager, Stanton Gray, Gregory A. Hawkins, Mario Medvedovic, Janice D. Wagner

**Affiliations:** 1 Center for Genomics and Personalized Medicine Research, Wake Forest School of Medicine, Winston-Salem, North Carolina, United States of America; 2 Department of Pathology, Wake Forest School of Medicine, Winston-Salem, North Carolina, United States of America; 3 Department of Environmental Health, University of Cincinnati, Cincinnati, Ohio, United States of America; 4 Center for Environmental Genetics, University of Cincinnati, Cincinnati, Ohio, United States of America; 5 Cincinnati Veteran Affairs Medical Center, Cincinnati, Ohio, United States of America; Florida State University, United States of America

## Abstract

Nutritional interventions are important alternatives for reducing the prevalence of many chronic diseases. Soy is a good source of protein that contains isoflavones, including genistein and daidzein, and may alter the risk of obesity, Type 2 diabetes, osteoporosis, cardiovascular disease, and reproductive cancers. We have shown previously in nonhuman primates that soy protein containing isoflavones leads to improved body weight, insulin sensitivity, lipid profiles, and atherosclerosis compared to protein without soy isoflavones (casein), and does not increase the risk of cancer. Since genistein has been shown to alter DNA methylation, we compared the methylation profiles of cynomolgus monkeys, from multiple tissues, eating two high-fat, typical American diets (TAD) with similar macronutrient contents, with or without soy protein. DNA methylation status was successfully determined for 80.6% of the probes in at least one tissue using Illumina's HumanMethylation27 BeadChip. Overall methylation increased in liver and muscle tissue when monkeys switched from the TAD-soy to the TAD-casein diets. Genes involved in epigenetic processes, specifically homeobox genes (*HOXA5*, *HOXA11*, and *HOXB1*), and *ABCG5* were among those that changed between diets. These data support the use of the HumanMethylation27 BeadChip in cynomolgus monkeys and identify epigenetic changes associated with dietary interventions with soy protein that may potentially affect the etiology of complex diseases.

## Introduction

Dietary soy has been proposed to be a “heart healthy” food supplement. The FDA approved a health claim for soy protein and soy-based food products, based largely on the evidence that soy consumption improves plasma lipid and lipoprotein concentrations and might reduce the risk of cardiovascular disease (CVD), yet does not appear to increase cancer risk [1]. Isoflavones, in particular genistein and daidzein, are abundant in soy protein and, as they are structurally similar to estradiol, can bind to both estrogen receptor (ER) α and β, but with greater affinity for ER β [2]. Depending on their concentration, the concentration of endogenous estrogen, and ER number and type (e.g., ER α or β), these isoflavones can act with estrogen-like activity (i.e., agonists) or act as estrogen antagonists [3]. Thus, isoflavones have the potential to be endocrine disrupters, especially if given during key periods of development or reproductive stage [4], [5]. Soy is commonly used in infant formulas, and the safety of these formulas in developing infants has been controversial [6], [7]. The mechanisms involved in soy's beneficial and potentially adverse effects are complex and poorly understood.

The “fetal basis of adult disease” hypothesis [8] postulates “that nutrition and other environmental factors during prenatal and early development influence cellular plasticity, thereby altering susceptibility to adult CVD, type 2 diabetes (T2D), obesity, and other chronic diseases” [9]. An abundance of literature in animal models suggests that prenatal or early exposure to either estrogenic compounds or genistein specifically affects later risk of obesity [10], congestive heart failure [11], immune function [12], reproductive effects in males [13] and females [14], and cancers of the reproductive system, such as breast and prostate [15]–[19].

There is a significant body of evidence demonstrating that environmental exposures, including diet, lead to epigenetic changes. These changes offer one mechanism for “fetal programming” that can affect the onset of diseases later in life. For example, i*n utero* exposure to dietary genistein modified coat color in inbred mice by methylation of a CpG island that regulates expression of the Agouti coat color gene [9], [20]. This *in utero* gene regulation also modified the risk of adult obesity in the offspring and persisted into adulthood. These studies indicate the potential for dietary genistein to affect chronic diseases in mice through an epigenetic pathway, namely DNA methylation. While these rodent studies have been helpful in establishing the relationship between diet and DNA methylation, we propose using a more relevant primate model, cynomolgus monkeys, where sex hormone levels and risk of disease follow the same disease risk profile as in humans [21].

We hypothesized that changes in DNA methylation contribute to the effects we and others have observed in humans and animals consuming soy-based diets [22]. The diets for this study had similar macronutrient content with the exception of protein source, which was either soy- or casein/whey-based. To better understand this mechanism, we have performed DNA methylation analysis with multiple tissues from cynomolgus monkeys eating one of two diets based on that of a typical North American. DNA microarrays designed for human DNA methylation studies (the Illumina HumanMethylation27 Beadchip) were used to examine DNA from tissues relevant to common, complex diseases to determine if these two diets contributed to epigenetic changes that may be involved in complex disease susceptibility.

## Materials and Methods

### Ethics Statement

Approval for all procedures in this study was obtained from the Wake Forest University (WFU) Animal Care and Use Committee (A08-219). In addition, an appropriate environmental enrichment SOP for this project was approved by the WFU NHP Environmental Enrichment committee. This included, but was not limited to: monkeys pair-housed the majority of the time, but single-housed temporarily for feeding and other experimental requirements; monkeys located to have auditory, visual, and olfactory stimulation from other monkeys in the room, and; monkeys received species-appropriate toys and manipulata rotated every two weeks at minimum. Animals in this project were fully under the care of veterinarians at the WFU School of Medicine in accordance with the standards incorporated in The Guide to the Care and Use of Laboratory Animals (1996). All experimental procedures were performed only with sedated animals and appropriate analgesia was administered. Animal welfare and steps were taken to ameliorate suffering in accordance with the recommendations of the Weatherall report (2006).

### Diets

Most rodent and nonhuman primate studies are done with animals fed commercial chows that contain low fat and cholesterol content, with the major portion of the protein from soy. This diet results in quite high serum concentrations of isoflavones [22]–[24] and is unlike most Western diets. We designed two diets that mimic the macronutrient content of the typical American diet (TAD), with 35% of calories from fat, but with differing protein sources [22]. One diet (TAD-casein, LabDiet® 5L0P) has casein and whey as the primary protein source and is nearly devoid of soy protein, more typical of the diet consumed by North Americans. The other diet (TAD-soy, LabDiet® 5L0R) contains soy as the protein source to mimic a high soy supplementation diet, similar to a diet in the human population consuming soy and tofu products or monkeys consuming standard chow. It provides the equivalent on a caloric basis to 180 mg/person/day of soy isoflavones, resembling the isoflavone content of chow consumed by almost all monkeys used in research [22], [23], [25]. The high soy protein chows likely do not model disease patterns of people in Western countries, who eat very limited amounts of soy protein, and thus have relatively low isoflavone concentrations.

### Study Design

**Figure 1:**
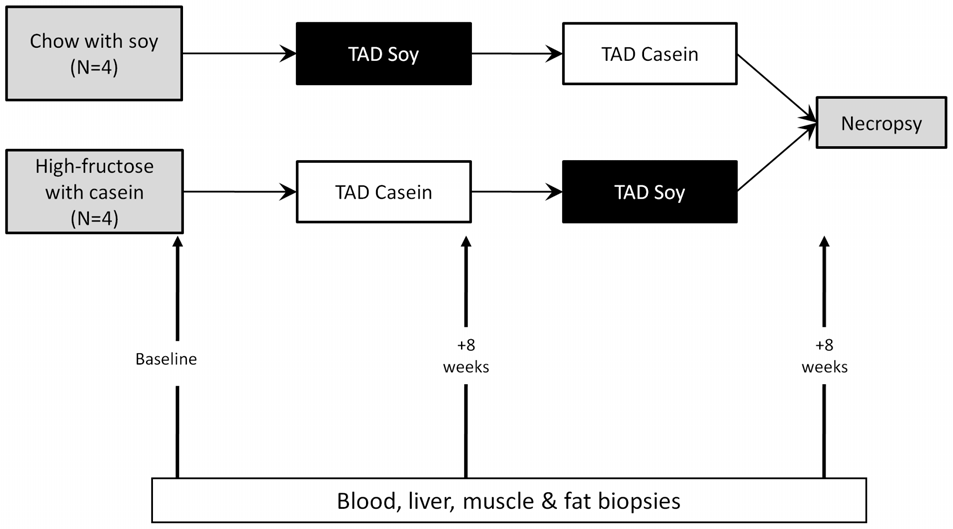
Study Design. Four cynomolgus monkeys were included in each diet arm. Measurements for clinical variables were performed after four weeks on each diet. DNA was isolated from blood, fat, liver, and muscle at the end of each diet period.

This study utilized monkeys that had been consuming two different diets at baseline, and then two diet changes were performed for each group of monkeys (Figure 1). Monkeys (N = 4 in each diet arm) were previously eating either standard monkey chow (with soy as the protein source) or a low cholesterol, high fructose diet with casein as the protein source [21]. Monkeys eating the standard chow diet (Figure 1, top) were switched to the TAD-soy diet due to their similar exposure to soy from chow (Diet Arm 1). These animals continued to eat the TAD-soy diet for eight weeks, at which time they were switched to the TAD-casein diet. The four monkeys eating the fructose-casein diet (Figure 1, bottom) were switched to the TAD-casein diet for 8 weeks, due to the similar exposure to casein, and then switched to the TAD-soy diet for an additional 8 weeks (Diet Arm 2). After four weeks of consuming each diet, blood samples were taken to determine plasma lipids and carbohydrate measures, and intravenous glucose tolerance tests (GTT) were done. Blood samples and fat, muscle, and liver biopsies were collected from all eight monkeys at baseline and at the end of each diet phase. As there was evidence for differences between monkeys in the two diet arms at baseline, physiologic measurements for TAD soy and TAD casein diets were evaluated within each arm using linear mixed models, adjusting for age of the animals and baseline measures. For all statistical comparisons, a two-sided p value <0.05 was considered significant. All procedures were approved by the Institutional Animal Care and Use Committee.

### Tissue Biopsies

Monkeys were sedated with ketamine HCl (15–20 mg/kg) intramuscularly. Aseptic surgical technique was used to collect subcutaneous adipose tissue, muscle, and liver. For subcutaneous adipose and muscle tissue, a 2 cm skin incision was made on the upper thigh and adjacent to the umbilicus, respectively. Subcutaneous adipose tissue over the abdomen was bluntly dissected, and quadriceps muscle was taken with a 6 mm punch biopsy, for approximately 0.25–0.50 g of tissue each. Liver samples were obtained by ultrasound-guided needle biopsies. A small, 2 mm skin incision was made caudal to the last rib on the right side of the ventral abdominal region. A 14-gauge liver biopsy needle was inserted through the skin incision to a depth of 1.5–3.0 cm. Ketoprofen (5 mg/kg, IM) and buprenorphine (0.01 mg/kg, IM) was given post-operatively for analgesia.

### DNA methylation analysis

DNA was isolated from blood, muscle, fat and liver after eight weeks on each diet. DNA was bisulfite-converted with the EZ DNA Methylation™ Kit (Zymo Research) and analyzed with Illumina Human Methylation27 BeadChips, which assay 27,578 human CpG sites from 14,000 highly annotated genes. To calculate the total DNA methylation proportions, only probes with a detection p-value less than 0.05, according to the program GenomeStudio (Illumina, Inc.), for all tissues was used. For all other analyses, probe-level data was normalized using quantile normalization and a gene-level methylation ratio was determined. Differential methylation between diets was determined using a linear model with a block effect.

Principal Component Analysis (PCA) was performed using data from all CpG loci with a detection p-value less than 0.05 in all monkeys (N = 8,002) and JMP Genomics software (SAS Institute, Cary, NC). Both loci (rows) and samples (columns) were centered to a mean zero prior to PCA determination.

### Sequencing and Pyrosequencing of Cynomolgus DNA

To obtain the DNA sequence of our differentially methylated genes in cynomolgus macaques, PCR and sequencing primers were designed by using a human/rhesus macaque consensus sequence. Sequencing was attempted around the seven CpG sites with significant changes between the two TAD diets, and four of these sites generated high-quality, unambiguous sequence. Pyrosequencing assays were designed for these four sites using PyroMark Assay Design software (Qiagen, Inc.). Pyrosequencing was performed on a Biotage PSQ96 instrument and data was analyzed with Pyro Q-CpG (Biotage, Inc.).

## Results

Monkeys eating two distinct protein diets were studied to delineate the effects of soy protein in animals initially fed standard monkey chow (which contains soy) or a casein-based diet (Figure 1). These monkeys had been consuming their initial diets for a minimum of two years before the current study, and as such, some phenotypic differences were observed between the two groups at baseline (Table 1). For this reason, the two diet arms were analyzed separately. Insulin resistance, as determined by HOMA-IR, was significantly higher in animals eating the initial high-fructose diet compared to animals eating chow (4.33 versus 3.00 HOMA units; p = 0.005). In addition, proinsulin levels were higher in the high fructose group (131.13 pmol/L versus 42.5 pmol/L, p = 0.025) and total plasma cholesterol was lower (91.75 mg/dL versus 102.25 mg/dL, p = 0.031). Over the course of the study, there were no significant changes in the clinical characteristics of monkeys in Diet Arm 1 (Table 1). In Diet Arm 2, fasting insulin levels were lower after changing from the TAD-casein to the TAD-soy diet (35.75 µU/ml versus17 µU/ml, p = 0.01). Related to the insulin levels, lower insulin resistance was also observed with the TAD-soy diet (2.67 versus 6.03 HOMA units, p = 0.02). No other clinical or physiologic parameters changed significantly between animals eating the different diets.

**Table 1:** Characteristics of monkeys on each diet at baseline and after diet changes.

	Diet Arm 1 (mean, SD)			
Baseline descriptives	Chow	TAD Soy	TAD Casein	P-value*
Body weight, kg	6.07 (0.68)	5.86 (0.92)	5.77 (0.63)	0.61
Fasting glucose, mg/dL	55.46 (5.73)	56.25 (2.22)	61.25 (3.95)	0.12
Fasting insulin, µU/mL	21.24 (13.6)	13.25 (6.34)	19 (10.1)	0.22
Insulin resistance index (HOMA units)	3 (2.08)	1.81 (0.98)	2.93 (1.72)	0.21
C-peptide, pg/mL	463.38 (226.52)	502.75 (296.91)	573 (235.42)	0.29
Proinsulin, pmol/L	42.5 (27.65)	38.25 (9.91)	45.25 (32.43)	0.57
Fructosamine, µmol/L	177.95 (12.22)	166.18 (12.35)	169.8 (8.06)	0.29
TPC, mg/dL	102.25 (12.28)	131.25 (31.63)	124.75 (45.51)	0.40
TG, mg/dL	85.75 (70.18)	67.75 (50.36)	61 (40.01)	0.71
HDL, mg/dL	46.75 (4.11)	63 (17.49)	62 (19.41)	0.62
CRP, ng/mL	657.32 (406.62)	637.98 (447.70)	933.2 (1121.92)	0.80

	Diet Arm 2 (mean, SD)			
Baseline descriptives	High Fructose	TAD Casein	TAD Soy	P-value*
Body weight, kg	8.4 (1.79)	7.36 (1.76)	7.49 (1.67)	0.59
Fasting glucose, mg/dL	66.69 (9.32)	68 (8.29)	62.75 (8.42)	0.27
Fasting insulin, µU/mL	**26.38 (11.76)**	**35.75 (7.23)**	**17 (2.58)**	**0.01**
Insulin resistance index (HOMA units)	**4.33 (1.98)**	**6.03 (1.7)**	**2.67 (0.74)**	**0.02**
C-peptide, pg/mL	296.25 (60.14)	352 (82.92)	303.75 (69.14)	0.34
Proinsulin, pmol/L	131.13 (35.86)	118.75 (41.19)	106.5 (31.94)	0.53
Fructosamine, µmol/L	170.7 (15.41)	155.25 (14.6)	168.9 (17.4)	0.10
TPC, mg/dL	91.75 (10.78)	121 (24.47)	128.75 (28.48)	0.73
TG, mg/dL	107.5 (24.56)	81 (26.55)	62 (16.87)	0.34
HDL, mg/dL	45 (6.98)	66.25 (19.31)	71.75 (9.54)	0.59
CRP, ng/mL	900.63 (186.94)	766.03 (279.24)	868.9 (271.96)	0.39

*adjusted for age and baseline values.

**Figure 2:**
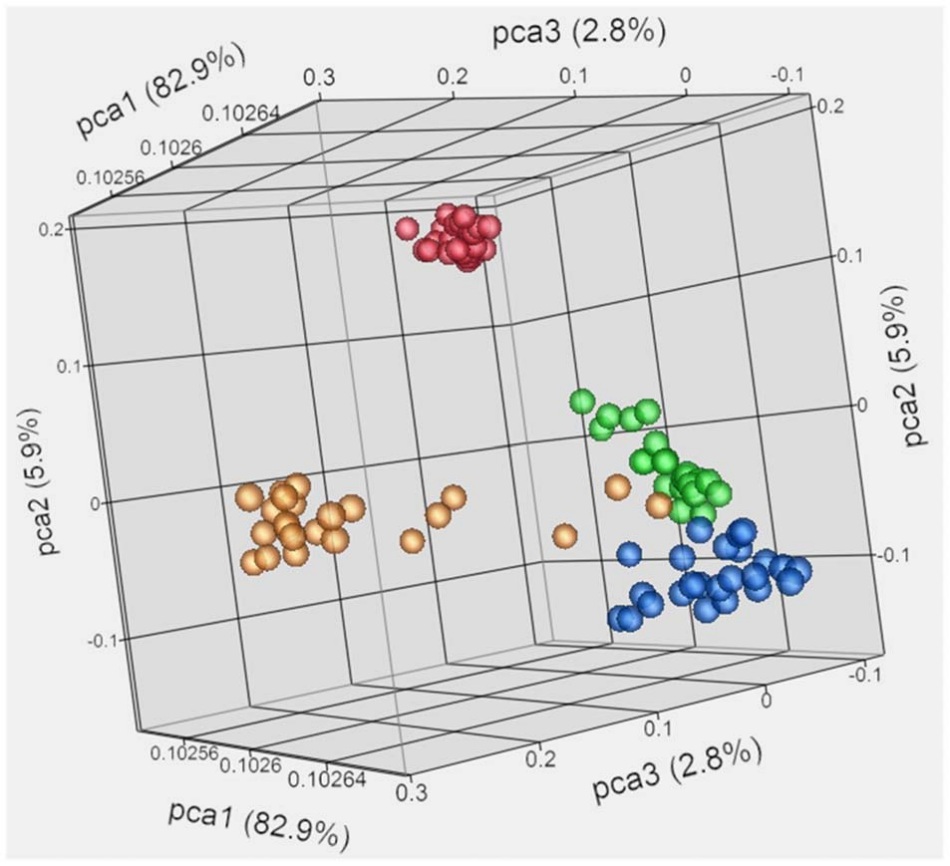
PCA analysis of DNA methylation levels from all monkeys eating each diet. Four clusters can be observed, distinguished by the tissue type. The axes are based on the eigenvalues of the three principal components determined. Tissues are differentiated by color: liver, gold; muscle, blue; fat, green; and blood, red.

We performed DNA methylation analysis using the HumanMethylation27 BeadChip (Illumina, Inc.). Using the “detection p-value” metric in GenomeStudio (which evaluates the probe intensity for each CpG site compared to a negative control), 80.6% of the assays generated a sufficient signal in at least one sample from cynomolgus monkeys. Using methylation data from all probes with a detection p-value less than 0.05, PCA analysis was performed with all monkeys, on each diet. Four clusters were observed, predominately defined by tissue type (Figure 2). Samples from blood provided the most compact cluster, indicating that the DNA methylation pattern from blood was highly similar among all samples. Six liver samples were outside of the liver cluster (three of these were from the same monkey), but all other samples formed easily observable clusters based on the tissue type (i.e., blood, liver, fat, or muscle). Using all animals and each diet condition, a total of 95 samples (one sample was unacceptable for analysis) were analyzed.

**Figure 3:**
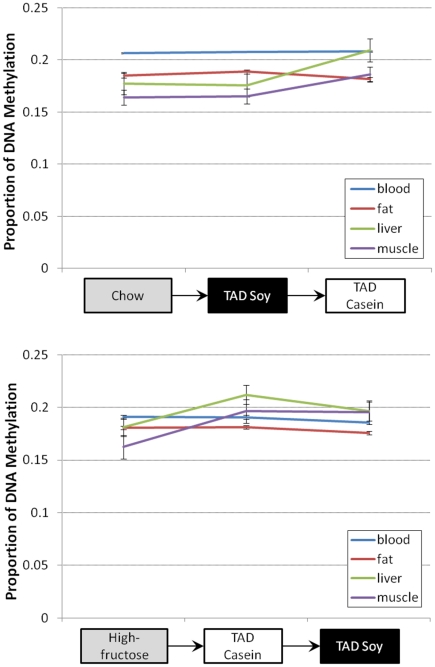
Overall proportion of DNA methylation in each tissue, by diet. Values are the means (with standard errors) of probes with a detection p-value <0.05 for all samples (N = 8,002) in each tissue. Four males were included in each diet group.

DNA methylation levels varied by diet and tissue type, and overall DNA methylation levels increased in liver and muscle after transitioning from the TAD-soy to the TAD-casein diet (Figure 3, top). The levels in blood remained the same while levels in fat decreased slightly. In liver, the proportion of overall DNA methylation increased from 0.175 to 0.209 (a 19% increase) and in muscle the increase was from 0.165 to 0.186 (a 13% increase) with the TAD-soy to TAD-casein change. Methylation also increased in liver and muscle after switching from the high-fructose casein diet to the high-fat TAD-casein diet (Figure 3, bottom). The proportion in liver increased from 0.181 to 0.212 (a 17% increase) and muscle increased from 0.162 to 0.197 (a 22% increase).

Gene-specific analyses were performed with a focus on the transition between the two TAD-based diets. Separate tissue-specific analyses were performed to identify changes at the p<0.01 and p<0.001 levels. Genes with p<0.01 were used to identify Gene Ontology (GO) and Kyoto Encyclopedia of Genes and Genomes (KEGG) pathways that were over-represented with each transition (Table 2). Significant pathways were identified from blood only with the TAD-soy to TAD-casein transition with Diet Arm 1; all tissues except muscle revealed significant pathways with the TAD-casein to TAD-soy change with Diet Arm 2.

**Table 2:** Pathways detected after TAD diet changes.

Diet Change	Tissue	No. genes with P<0.01	No. genes with P<0.001	Over-represented GO and KEGG pathways*	FDR P-value
**TAD-soy to TAD-casein**	Blood	137	3	preassembly of GPI anchor in ER membrane	0.030
				GPI anchor metabolic process	0.067
				GPI anchor biosynthetic process	0.067
				protein amino acid lipidation	0.076
				histone methyltransferase activity	0.076
				lipoprotein biosynthetic process	0.082
				P-P-bond-hydrolysis-driven protein transmembrane transp. act.	0.082
				macromolecule transmembrane transporter activity	0.082
				protein methyltransferase activity	0.089
	Fat	212	18	No associated pathways	NA
	Liver	359	25	No associated pathways	NA
	Muscle	677	82	No associated pathways	NA
**TAD-casein to TAD-soy**	Blood	126	5	autophagic vacuole	0.059
	Fat	264	22	embryonic skeletal system development	0.0068
				cranial nerve development	0.011
				skeletal system morphogenesis	0.028
				proton-transp. two-sector ATPase complex proton-transp. domain	0.039
				forelimb morphogenesis	0.044
				multi-organism process	0.044
				striated muscle cell proliferation	0.054
				nerve development	0.054
				embryonic skeletal system morphogenesis	0.054
				cardiac muscle cell proliferation	0.054
				proton-transporting ATP synthase complex coupling factor F(o)	0.099
	Liver	69	3	anion:cation symporter activity	0.00012
				hydro-lyase activity	0.0011
				carbon-oxygen lyase activity	0.019
				protein kinase A binding	0.053
				carbonate dehydratase activity	0.068
	Muscle	189	17	No associated pathways	NA

*All genes with p<0.01 were used to identify GO and KEGG pathways.

DNA methylation levels for seven individual genes changed significantly (False Discovery Rate, FDR, p<0.20) after switching diets – four with the TAD-soy to TAD-casein transition and three with the TAD-casein to TAD-soy diet change (Table 3). These genes included homeobox genes (*HOXA5*, *HOXA11*, *HOXB1*) and ATP-binding cassette, sub-family, member 5 (*ABCG5*). The sites for *HOXA5* and *HOXA11* are separated by ∼43 kb, suggesting that this entire genomic region may be epigenetically marked. No genomic sequence for cynomolgus macaques is currently available, so to further investigate these findings the region around ten of the 50bp probes from these seven genes was sequenced to determine the identity between the human and cynomolgus sequences. Identities between human and cynomolgus DNA sequences ranged from 0.94 to 1.0, suggesting that these specific probes were assaying the correct CpG loci of the respective human sites.

**Table 3:** Genes with significant DNA methylation changes (FDR P<0.20) after the diet switch.

Diet	Tissue	Gene Symbol	Illumina Probe ID	Methylation Difference	P-value	FDR P-value
TAD-soy to TAD-casein	muscle	*HOXA5*	cg02248486	−0.25	7.06E-06	0.087
	muscle	*HOXA11*	cg15760840	−0.47	7.47E-06	0.087
	muscle	*NTRK3*	cg26232187	0.26	1.00E-05	0.087
	muscle	PLA2G12A	cg24974477	−0.041	2.09E-05	0.14
TAD-casein to TAD-soy	fat	*ABCG5*	cg25781162	−0.17	1.35E-06	0.035
	fat	*HOXB1*	cg07823492	0.17	4.45E-06	0.058
	fat	*TBX5*	cg21907579	0.22	9.04E-06	0.078

**Figure 4:**
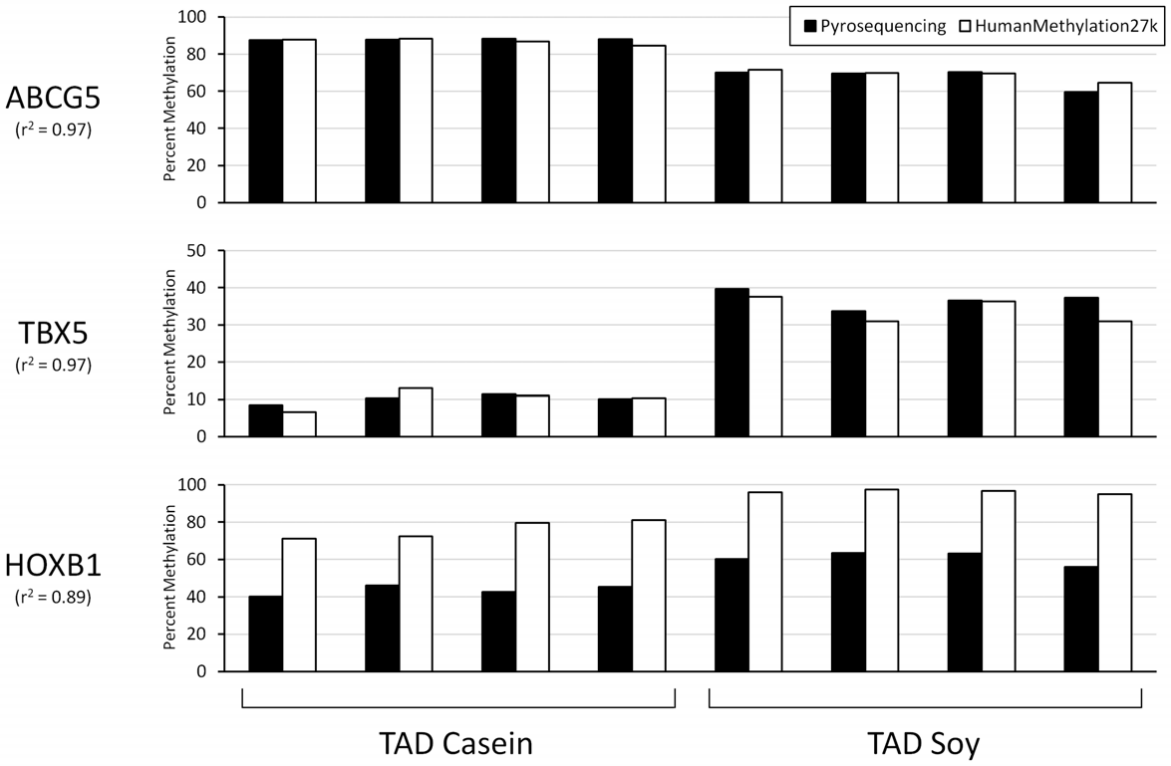
Comparison of HumanMethylation27 BeadChip with pyrosequencing. The gene associated with each CpG site is shown to the left of each graph. The correlation (r^2^) between pyrosequencing (black bars) and the HumanMethylation27 BeadChip (white bars) is shown under each gene symbol. The four pairs of bars on the left of each graph represent the percent DNA methylation from monkeys eating the TAD Casein diet, and the four pairs on the right half represent the percent DNA methylation from monkeys eating the TAD Soy diet.

To validate the HumanMethylation27 BeadChip data, we performed pyrosequencing of four of the top seven CpG sites with high-quality sequence data from cynomolgus monkeys. Data from three of these sites (*ABCG5*, *TBX5*, and *HOXB1*) were highly correlated (Figure 4). For sites in *ABCG* and *TBX5*, the data were virtually identical between the two assays (r^2^ = 0.97 for both), which is consistent with the 100% identity between the human and cynomolgus sequences that encompass the 50bp Illumina probes. The correlation was lower for *HOXB1* (r^2^ = 0.89), where a single base difference exists between the human probe and cynomolgus monkey sequences. Since the base difference is a “C” in cynomolgus monkeys and a “T” in humans, this difference has no effect after bisulfite conversion, where both bases would become a “T”. A second sequence difference 2bp 3′ of the probe creates an adjacent CpG in the cynomolgus monkeys that is absent in humans. This change would alter the fluorescent nucleotide extended in the assay and therefore affect the signal. Importantly, this change alters the apparent magnitude of the methylation but does not affect the difference between samples. A fourth site examined was located in *HOXA11*, where a significant difference was detected in muscle tissue (Table 3). Unfotunately, we did not have sufficient DNA from muscle, so DNA from fat tissue was used for the validation assay. This site had the lowest correlation of the four tested (r^2^ = 0.74), most likely due to a single base difference between the cynomolgus monkey and human sequences. This change (a “C” in humans is a “G” in cynomolgus monkeys), which is located at a CpG site within the Illumina probe, would still differ after bisulfite conversion, decreasing the affinity of the probe for the genomic DNA.

## Discussion

To elucidate the genetic and molecular mechanisms of complex diseases and disentangle the age- and environment-related factors, relevant animal models are needed. One limitation of animal models, specifically nonhuman primates, is that the current genomic tools have been developed primarily for humans, where well-annotated sequence data is easily available. This pilot study was done to determine the usefulness of the HumanMethylation27 BeadChip (Illumina, Inc.) in cynomolgus monkeys. In addition, we characterized tissue-specific changes in DNA methylation that occurred after altering diets that differed in protein content. DNA methylation data was easily distinguished by tissue type using PCA, supporting the ability of the HumanMethylation27 BeadChip to properly assay the cynomolgus genome. Overall DNA methylation levels increased in liver and muscle tissues when animals were switched to the TAD-casein diet. Gene specific changes in specific pathways, such as those involved in epigenetic regulation, were also identified. Significant DNA methylation changes in seven individual genes from muscle and fat samples were also observed after diet changes, and four of these were validated using an alternative assay.

DNA methylation changes were detected after the relatively short dietary study periods, most notably in liver and muscle. It is unclear why liver and muscle demonstrated the largest changes in DNA methylation, but it may be due to the effect of soy components (e.g., isoflavones) on these tissues specifically. Based on our previous studies comparing the effects of dietary soy versus casein in cynomolgus monkeys, changes in triglyceride levels and glycemic control were noted [22]. These changes are consistent with variable methylation in genes in liver and muscle, as observed in this study. Also consistent is that measures of insulin and insulin sensitivity, which are affected by the ability of peripheral muscles to respond to insulin, were the only phenotypic traits significantly different in this study with the transition from the TAD-casein to the TAD-soy diet (Table 1).

Genes with changes in DNA methylation were more likely to be found in specific GO and KEGG pathways (Table 2). Some of these pathways, such as histone methyltransferase activity and protein methyltransferase activity, are directly related to epigenetic modifications. Other processes appear to be more tissue-specific. Gene-specific analysis identified seven individual genes that changed significantly after the diet changes – four after the transition from TAD-soy to TAD-casein and three after transitioning from TAD-casein to TAD-soy. All four of the genes from Diet Arm 1 were from muscle tissue and included two homeobox genes, *HOXA5* and *HOXA11*, which are located approximately 37 kb apart on chromosome 7. For both of these genes there was a decrease in methylation when the monkeys changed from the TAD-soy to the TAD-casein diet, consistent with an overall decrease of DNA methylation of this entire genomic region. Homeobox (Hox) genes encode a unique set of transcription factors that play key roles in development. More recently, epigenetic modification of Hox genes, in general, has been shown to be important in a variety of cancers. The other gene of interest with a significant change was *ABCG5*, which functions to limit intestinal absorption and promote biliary excretion of sterols. In fact, mutations in this gene cause the autosomal recessive disease sitosterolemia, which is characterized by markedly increased absorption of sterols by the intestine and a limited ability to secrete sterols into bile [26]. Individuals with this condition are at an increased risk of developing atherosclerosis and coronary artery disease. Notably, dietary soy consumption has been shown to lower plasma lipids and decrease the progression of atherosclerosis [1].

There is a significant body of evidence demonstrating that environmental exposures, including diet, alter important DNA methylation patterns, resulting in “fetal programming” that may affect adult disease. For example, maternal nutrition prior and during gestation, and during lactation, plays an essential role in the establishment of epigenetic patterns in the offspring. Individuals who were prenatally exposed to famine during the Dutch Hunger Winter in 1944-45 had, six decades later, less DNA methylation of the paternally imprinted *IGF2* gene compared with their unexposed, same-sex siblings [27]. The effects of genistein has been examined in rodents, where gene- and tissue-specific DNA methylation changes have been observed [20], [28], [29]. Here, we show changes in primate gene methylation associated with soy diets containing genistein, consistent with these rodent studies.

There was a significant decrease in fasting insulin and HOMA index values in animals that transitioned from the TAD-casein to the TAD-soy diet, suggesting improved insulin sensitivity with the TAD-soy diet. There was a complementary increase (although not statistically significant) with these same measures in animals that transitioned from the TAD-soy to TAD-casein. No significant change in body weight was observed in monkeys in either diet arm, likely due to the short study periods and the small number of animals studied. In studies with longer diet interventions, monkeys consuming TAD-soy diets had lower body weight in addition to improved lipids and measures of glycemic control [22]

We and others have previously reported significant variation in complex disease phenotypes that appears to be mediated by dietary soy (e.g., [22], [30]–[32]). To study this variability systematically, we have leveraged the available molecular tools designed for the human genome with nonhuman primates, which have a high degree of DNA identity with humans, but can be studied in a controlled environmental setting (including diet). We identified significant, tissue-specific changes in DNA methylation due to diet changes. These data provide evidence that dietary soy affects global DNA methylation, which may contribute to soy's beneficial effects on multiple complex phenotypes. Additional studies in nonhuman primates and humans are necessary to determine the long-term physiologic and potentially pathologic consequences of soy and other dietary supplements on epigenetic regulation and human health.
